# 

**Published:** 1893-12-16

**Authors:** 


PRICE TWOPENCE.

No. 377, Vol. XV.—Dec. 16th, 1893.

Registered at the General Post Office as a Newspaper for transmission in the United Kingdom and Abroad.]

December 16th, 189[illegible]

WITH EXTRA NURSING SUPPLEMENT

Per Annum, 8s. 6d., post free.

Monthly Parts, 6d.; post free, 8d.

Ditto per Annum, 8s., post free.

### THE HOSPITAL

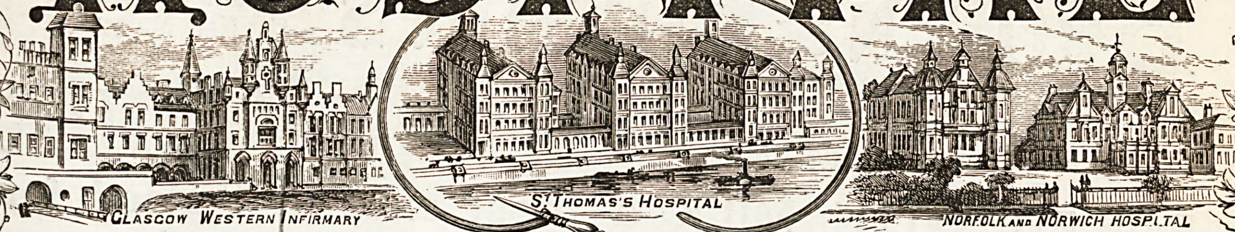

WITH EXTRA NURSING SUPPLEMENT.

No. 377. Vol. XV. Saturday, December 16th, 1893. [Twopence.

